# Epidemiologic Insights into Stone Disease as a Systemic Disorder

**DOI:** 10.1063/1.2723564

**Published:** 2007-04-05

**Authors:** Gary C. Curhan

**Affiliations:** Renal Division, Brigham and Women’s Hospital, Harvard Medical School, 181 Longwood Avenue, Boston, MA; Channing Laboratory, Department of Medicine, Brigham and Women’s Hospital, Harvard Medical School, 181 Longwood Avenue, Boston, MA; Department of Epidemiology, Harvard School of Public Health, 181 Longwood Avenue, Boston, MA; Indiana University School of Medicine; Methodist Hospital Institute for Kidney Stone Disease; Indiana University School of Medicine

## Abstract

Examining the epidemiology of stone disease can provide insight into etiology. There is a growing body of evidence that stone disease is not simply a disorder of the kidney. In fact, nephrolithiasis is clearly a systemic disorder. Conditions associated with stone disease include the classic ones such as inflammatory bowel disease and primary hyperparathyroidism. More recent studies have demonstrated strong associations with obesity, gout, diabetes and hypertension. Future studies will help uncover the underlying common pathophysiologic abnormalities.

## INTRODUCTION

Epidemiology is defined as the study of the distribution and determinants of disease. Examination of these factors may provide insight into the systemic nature of nephrolithiasis.

The distribution of nephrolithiasis is usually quantified in terms of prevalence and incidence. In the US, the prevalence of a history of stone disease increased from 3.8% in NHANES II (1976-80) to 5.2% in NHANES III (1988-1994) [1]. Somewhat surprisingly, a recent study from the Olmstead County Epidemiology Project reported that incidence rates appear to have stabilized or may have fallen over the past two decades [2].

### Dietary risk factors

Risk factors for stone disease are usually divided into three main categories: dietary, non-dietary and urinary. It is important to note that the importance of different risk factors varies by age and sex (and likely race).

Dietary factors that have been consistently shown to reduce the risk of stone formation include higher dietary calcium intake and higher fluid intake [3-5]. In older men and women, higher potassium intake is also associated with reduced risk of stone formation. In women, but not in men, higher phytate intake reduces the risk. Sucrose intake is associated with an increased risk of stone formation. In older men, higher

CP900, *Renal Stone Disease, 1st Annual International Urolithiasis Research Symposium,*
edited by A. P. Evan, J. E. Lingeman, and J. C. Williams, Jr.
© 2007 American Institute of Physics 978-0-7354-0406-9/07/$23.00

vitamin C intake increases the risk. Other factors such as animal protein and magnesium have not been consistently associated with risk.

Individual beverages may impact risk independent of their effect on urine volume [6, 7]. Beverages that have been associated with a reduced risk of stone formation include milk, coffee, tea, beer and wine, likely by increasing urine flow and reducing urine concentration. Grapefruit juice appears to increase the risk but the mechanism is unknown.

## Non-dietary risk factors

A number of systemic conditions have been known to increase the risk of stone formation including inflammatory bowel disease and primary hyperparathyroidism. However, only recently has it been appreciated that more common systemic disorders influence the risk of nephrolithiasis. These conditions include obesity [8], gout [9, 10], and diabetes [11].

### *Obesity*

While obesity has long been believed to be associated with stone formation, evidence was lacking until recently. Taylor and colleagues [8] found in a prospective study of three large cohorts (Nurses' Health Study I, Nurses' Health Study II and the Health Professionals Follow-up Study) that the risk of stone formation increased with increasing weight (Fig. 1); the magnitude of the increase was greater in women than in men.

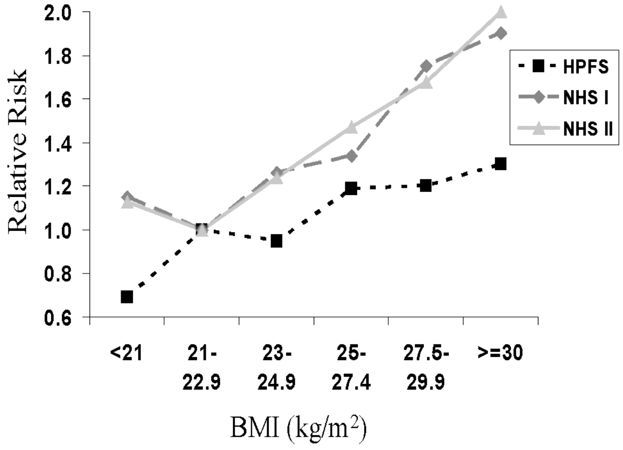

**Figure 1.** Relative risk of symptomatic kidney stones versus body mass index [8]. HPFS: Health Professionals Follow-up Study. NHS: Nurses' Health Study.

Similar relations were seen when examining other measures of body size such as weight and waist circumference. These associations were independent of age, fluid intake and other dietary risk factors. Thus, it appears that being overweight or obese increases the risk of stone formation independent of dietary factors.

### *Gout*

Gout has long been associated with risk of uric acid stones but may also increase the risk of calcium oxalate stones. Using data from NHANES III, gout was associated with a 46% increased risk of being a stone former in men and 65% increased risk in women [10]. In the one prospective study, men with a history of gout were more than twice as likely as those with no history of gout to subsequently develop a kidney stone, independent of age, dietary factors and body mass index [9].

### *Diabetes mellitus*

Insulin resistance may be one potential explanation for the increased risk of stone formation observed with increasing BMI. The exact roles of insulin and insulin resistance in stone formation remain unclear. The association between a history of type II diabetes mellitus and risk of nephrolithiasis has only recently been studied. In cross-sectional analyses, men and women with a history of diabetes were more likely to also have a history of nephrolithiasis [11]. When examined prospectively, the risk was increased by 58% in younger women and 27% in older women; there was no association in men but this may have been due to the late mean age of onset of diabetes in the male cohort.

### *Hypertension*

A history of nephrolithiasis increases the risk of subsequently developing hypertension [12, 13]. However, a history of hypertension does not appear to increase the risk of stone formation. One possible explanation is that hypertension is more likely to develop as a result of renal damage related the obstruction or treatment of stone disease (e.g. ESWL).

## Urinary risk factors

Examination of 24-hour urine results may provide insight into the potential mechanisms that will explain the associations between systemic disorders and nephrolithiasis. To date, two large studies have examined the relation between body size and 24-hour urine composition [14, 15]. Maalouf and colleagues reported an inverse association between urine pH and body weight [15]. Taylor and colleagues reported on all the commonly measured urinary factors [16]. In addition to confirming the inverse association with urine pH, an inverse association was observed for urinary calcium in women. Positive associations were seen for urine oxalate and citrate in women, but not men, and urine uric acid. There was no association with total volume.

The relation between 24-hour urine composition and risk of hypertension has also been studied [17]. Surprisingly, there was no consistent association in a cross-sectional study between history of hypertension and urine excretion of calcium, magnesium, sodium and potassium. However, urine citrate was strongly inversely associated with hypertension. The role of acid-base regulation in the etiology of hypertension deserves further study.

## CONCLUSIONS

Nephrolithiasis is clearly a systemic disorder and a variety of factors influence urine composition of lithogenic substances, including the generation of acid and oxalate. Well accepted dietary risk factors, such as dietary calcium, are influenced by age, sex and body mass index. Future studies need to examine how these systemic factors interrelate and should result in improved preventive strategies that can be tailored to the individual patient.

## ACKNOWLEDGMENTS

This work was supported by grants from the National Institutes of Health including Grants DK59583, DK73381, CA87969, CA55075, and CA50385.
